# A primitive neuroectodermal tumor in an adult: Case report of a unique location and MRI characteristics

**DOI:** 10.1097/MD.0000000000009933

**Published:** 2018-02-16

**Authors:** Xin He, Zhongping Chen, Yutong Dong, Dan Tong

**Affiliations:** a Department of Radiology; b Department of Gastroenterology, The First Hospital of Jilin University, Changchun, Jilin, China.

**Keywords:** adult, brain tumor, malignant, primitive neuroectodermal tumor, ventricle

## Abstract

**Rationale:**

Central nervous system primitive neuroectodermal tumors (CNS PNETs) mostly occur in children and present as cerebellar medulloblastoma. A few cases of PNETs occur in the cerebral hemisphere. The presence of a PNET in ventricles is extremely rare. The prognosis of CNS PNET is extremely poor, and the 5-year survival rate does not exceed 35%. In the present study, we describe the first case of a PNET in the ventricles with good prognosis.

**Patient concerns:**

The case of a 36-year-old man is reported, who presented with a progressively worsening headache for 2 months.

**Diagnoses:**

Magnetic resonance imaging (MRI) revealed multiple tubercula on the walls of the lateral and third ventricles. Histopathologic analysis revealed a hypercellular tumor with small round cells containing hyperchromatic nuclei and a high nucleus:cytoplasm ratio. The analysis was consistent with PNET.

**Interventions:**

Radiation therapy covering the entire craniospinal axis was administered, with Temozolomide for synchronous auxiliary treatment.

**Outcomes:**

The patient was follow-up for a year and showed no signs of recurrence.

**Lessons:**

We present the first CNS PNET located in the ventricles with good prognosis. In this case, radiotherapy with Temozolomide auxiliary treatment presented good efficacy and safety to treat PNET. Additional studies on biomarkers may be useful in predicting personalized therapeutic response.

## 1 Introduction

Primitive neuroectodermal tumor (PNET) originates from primitive neuroepithelial cells and occurs in the central nervous system (CNS) and the surrounding muscles, bones, and organization. PNET is a primitive, undifferentiated small round cell malignant tumor. On the basis of different sites of the tumor, PNET is divided into central and peripheral types.^[1]^ Central PNET mostly occurs in children and accounts for only 1% of primary CNS tumors. Ventricular primary PNET is extremely rare; to the best of our knowledge, only 4 cases of recurrence have been reported in the English literatures ^[2–5]^ In this study, we present the first case of the PNET occurring in a 36-year-old man in his ventricles. The standard treatment for CNS PNETs consists of major surgical resection when feasible, followed by radiotherapy and chemotherapy, but survival rates are consistently lower in spite of multimodal therapy. In our case, the patient obtained 1-year progression-free survival by radiotherapy and chemotherapy. This is the first case that highly responds to radiotherapy and chemotherapy.

## 2 Case report

**Figure 1:**
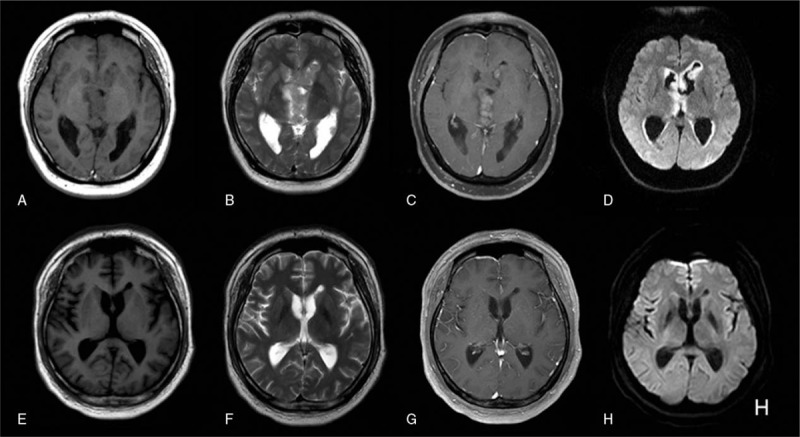
(A∼D) before radiotherapy and chemotherapy, the MRI revealed multiple tubercule on the wall of lateral and third ventricle, which were hypointense on T1-weighted images, hyperintense on T2-weighted images, and hyperintense on diffusion-weighted images (DWIs). (E∼H) After 1 month remedy, MRI showed completely disappear of the tumor.

**Figure 2:**
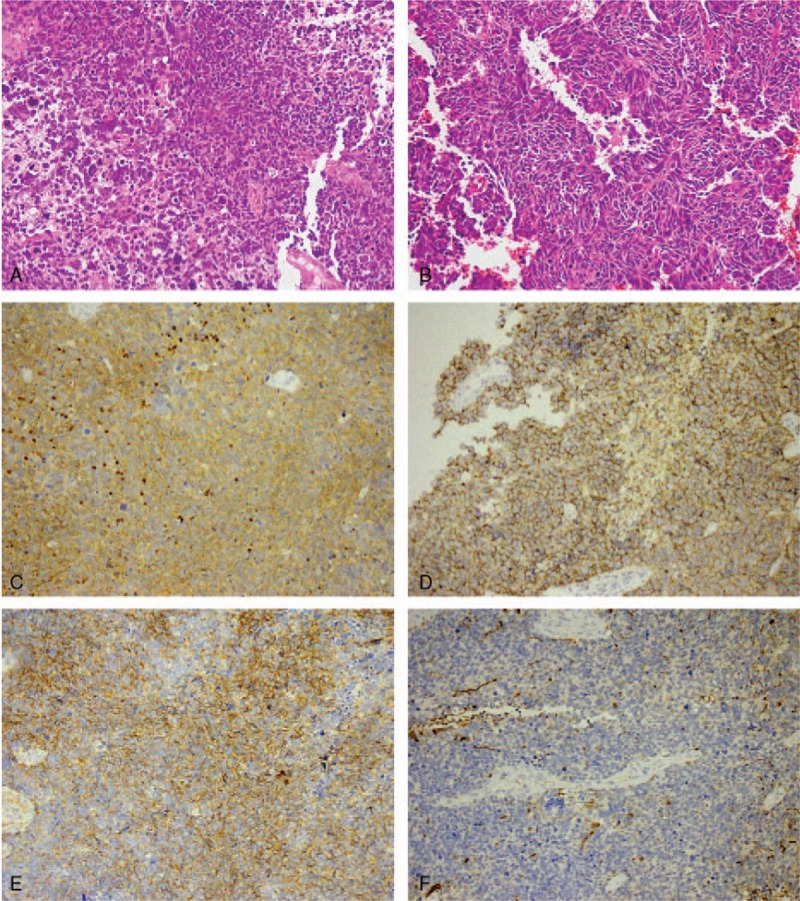
(A) H&E-stained biopsy revealed that this tumor was hypercellular with small round cells containing hyperchromatic nuclei and a high nucleocytoplasmic ratio. (B) High magnification shows cell clusters around a fibrinoid matrix (Homer–Wright rosettes). (C∼F) Immunohistochemical examination showed positive reaction for NSE, CD56, CD99, and s-100, respectively.

This is a case report and informed consent was obtained from the patient. A 36-year-old male presented to our hospital complaining of a headache, dizziness, and forgetfulness without an obvious cause. There were no sensory or cranial nerve deficits. Magnetic resonance imaging (MRI) of the brain revealed multiple nodules on the wall of the bilateral and third ventricles. The nodules were hypointense on T1-weighted images and hyperintense on T2-weighted images, with high signal on diffusion-weighted images (DWIs) (Fig. 1A–D). Postcontrast T1-weighted image showed homogeneously enhancement. Because of the depth of the lesion site and the vicinity of complex neurovascular structures, surgical access to lesions of petroclival region represented a challenge. So, this patient underwent a biopsy. Histologically, the tumor was hypercellular with small round cells containing hyperchromatic nuclei with a high nucleus:cytoplasm ratio (Fig. 2 A and B). Many cells contained irregular nuclei. According to immunohistochemical analysis, the tumor was positive for neuron-specific enolase (NSE), CD99, CD56, and S-100 expression (Fig. 2C–F) and negative for Synaptophysin (Syn), Vimentin, and epithelial membrane antigen (EMA) expression. The Ki-67 index was 70%. On the basis of these findings, this tumor was diagnosed as a PNET.

Next, radiation therapy covering the entire craniospinal axis was administered. On the third day of radiation therapy, 160 mg/day temozolomide was administered for synchronous auxiliary treatment. Radiotherapy of the entire craniospinal axis was performed 20 times. A followed MRI showed that the tumor had disappeared (Fig. 1 E–H). Further definitive management consists of cerebral local intensity modulated treatment; however, radiotherapy was suspended after the third iteration because of thrombocytopenia, so the patient received leukocyte and thrombopoietin injections. After the patient's thrombocyte count stabilized, the whole radiotherapy plan was completed. Meanwhile, temozolomide auxiliary treatment was also suspended. The patient underwent follow-up for a year and showed no signs of recurrence.

## 3 Discussion

PNET, a term coined in 1973 by Hart and Earle, is a malignant tumor that originates from primitive neuroepithelial cells and occurs in the CNS and the surrounding muscles, bones, and organization.^[5]^ Clinically, intracranial PNET is common in children but rarely occurs in adults, accounting for only 0.46% of adult cranial tumors.^[3]^ Intracranial PNET is often located in the cerebral hemisphere. Ašmonienė et al^[3]^ reviewed the literature and described 57 adult PNETs. The tumor is uniformly distributed in the frontal lobe, temporal lobe, and parietal lobe, and is greater than 6 cm in size. To our knowledge, only 4 cases in ventricles have been reported in the literature, but lesions in those cases presented as isolated solid mass. Patients with multiple lesions in the ventricles have not been reported in the previous published literatures. In our case, the tumor has multiple lesions and was distributed along the ventricular wall, possibly related to the residual neuroectodermal cells near the ventricle. These cells did not differentiate into normal neurons, leading to a “primitive” tumor. The clinical manifestations are associated with the site of the tumor, which is often characterized by high cranial pressure, and may be associated with epilepsy, abnormal mental disorders, and mild hemiplegia.^[6,7]^

Pathologically, PNET is densely cellular with undifferentiated, small, and hyperchromatic small round cells. The nuclei are short and round or spindle-shaped with less cytoplasm. Mitosis is usually observed. The characteristic feature is “Homer-Wright rosettes.”^[8,9]^ Immunohistochemical examination PNET may show glial, neuronal, and ependymal direction differentiation or the coexistence of multiple directions of differentiation.^[10]^

According to several published reports, the MRI features of AMFB have been summarized. Typically, the mass reveals a low signal intensity on T1-weighted image, and high signal intensity on T2-weighted image. The mass shows acidly enhancement after contrast MRI scan. The tumor signal is not uniform, mostly due to necrosis and cystic changes, or accompanied by hemorrhage and calcification in the tumor. Although the location of this PNET was unique, the MRI signal characteristics of this case were similar to those of tumors occurring in the brain hemisphere, such as nonperitumoral edema, and well-defined borders. One possible reason for these characteristics is that the main mechanism of PNET growth is proliferation, which is different from the infiltrative growth observed in other malignant brain tumors. The enhanced extent of the tumor was associated with the number of blood vessels in the tumor.^[11]^ The solid composition in DWI can help identify the solid component of the tumor.^[12]^ In this case, the tumor had high signal in DWI and obviously enhanced, which shows more densely arranged tumor cells with a rich blood supply, which has certain significance in differential diagnosis.

A treatment strategy for PNETs has not yet been established. In accordance with the patient's clinical status, maximum resection of the tumor, chemotherapy, and radiotherapy were performed as standard procedures.^[3]^ However, in our case, because of the special location, the treatment is different from the traditional strategies for the tumor could not be removed by surgery. Radiation of the entire craniospinal axis has also been recommended on the basis of CNS dissemination risk and improved prognosis. Gaffney recommends radiotherapy at a dose of 50 to 55 Gy for the tumor site for 7 to 8 weeks, which is in accordance with our plan.^[13]^ Note that the tumor in our case was in the ventricles, making it easier to disseminate through the cerebrospinal fluid to the whole brain and spinal canal. So, radiotherapy of the entire craniospinal axis was administrated. Currently, the role of chemotherapy in CNS PNET treatment is controversial.^[5,14,15]^

The 5-year survival rate of adults with PNET is less than 35%.^[3,5,16]^ Nonetheless, the prognosis of patient in our case is very good, because the tumor was highly sensitive to radiation and chemotherapy. Some studies discovered some molecular markers affecting resistance against radiation therapy, and Rad51 is one of these key proteins. Tumors with high expression of either LIN28 or OLIG2 or elevated level of Rad51 were significantly associated with poorer prognosis. Predictive biomarkers could discriminate patients who are most likely to be sensitive to radiotherapy and avoid unnecessary surgery. Thus, additional studies on biomarkers may be useful in predicting personalized therapeutic response.^[14–20]^

## References

[1] Prasad AN Supratentorial PNET in a young child. Indian J Pediatr 2011;78:613–5. 21213074 10.1007/s12098-010-0301-0

[2] Lester RA Brown LC Eckel LJ Clinical outcomes of children and adults with central nervous system primitive neuroectodermal tumor. J Neurooncol 2014;120:371–9. 25115737 10.1007/s11060-014-1561-8

[3] Ašmonienė V Skiriutė D Gudinavičienė I A primary primitive neuroectodermal tumor of the central nervous system in a 51-year-old Woman: a case report and literature review. Medicina (Kaunas) 2011;47:440–5. 22123553

[4] Chao Y Jie B Kai W MRI features of primitive neuroectodermal tumor of the head at uncommon locations. Chin J Med Imaging Technol 2012;28:640–3.

[5] Espino Barros Palau A Khan K Morgan ML Suprasellar primitive neuroectodermal tumor in an adult. J Neuroophthalmol 2016;36:299–303. 26517622 10.1097/WNO.0000000000000312

[6] Papadopoulos EK Fountas KN Brotis AG A supratentorial primitive neuroectodermal tumor presenting with intracranial hemorrhage in a 42-year-old man: a case report and review of the literature. J Med Case Rep 2013;7:86. 23537064 10.1186/1752-1947-7-86 PMC3623814

[7] Ohba S Yoshida K Hirose Y A supratentorial primitive neuroectodermal tumor in an adult: a case report and review of the literature. J Neurooncol 2008;86:217–24. 17713720 10.1007/s11060-007-9466-4

[8] Chawla A Emmanuel JV Seow WT Paediatric PNET: pre-surgical MRI features. Clin Radiol 2007;62:43–52. 17145263 10.1016/j.crad.2006.09.008

[9] Krampulz T Hans VH Oppel F Long-term relapse-free survival with supratentorial primitive neuroectodermal tumor in an adult: a case report. J Neurooncol 2006;77:291–4. 16528456 10.1007/s11060-005-9041-9

[10] Pigott TJ Punt JA Lowe JS The clinical, radiological and histopathological features of cerebral primitive neuroectodermal tumours. Br J Neurosurg 1990;4:287–97. 2171558 10.3109/02688699008992738

[11] Pickuth D Leutloff U Computed tomography and magnetic resonance imaging findings in primitive neuroectodermal tumours in adults. Br J Radiol 1996;69:1–5. 10.1259/0007-1285-69-817-1 8785615

[12] Shigu T Kagawa T Kimura Y Supratentorial primitive neuroectodermal tumor in an aged patient. Neuro Med Chir (Tokyo) 2005;45:530–5. 10.2176/nmc.45.530 16247240

[13] Batsakis JG Mackay B el-Naggar AK Ewing's sarcoma and peripheral primitive neuroectodermal tumor: an interim report. Ann Otol Rhinol Laryngol 1996;105:838–43. 8865780 10.1177/000348949610501014

[14] Raghuram CP Moreno L Zacharoulis S Is there a role for high dose chemotherapy with hematopoietic stem cell rescue in patients with relapsed supratentorial PNET? J Neurooncol 2012;106:441–7. 21850536 10.1007/s11060-011-0690-6

[15] Cefalo G Massimino M Ruggiero A Temozolomide is an active agent in children with recurrent medulloblastoma/primitive neuroectodermal tumor: an Italian multi-institutional phase II trial. Neuro Oncol 2014;16:748–53. 24482446 10.1093/neuonc/not320 PMC3984557

[16] Louis DN Hochberg FH Cerebral primitive neuroectodermal tumor in an adult, with spinal cord metastasis after 18-year dormancy. J Neurooncol 1990;9:77–80. 2170592 10.1007/BF00167072

[17] Biswas A Mallick S Purkait S Treatment outcome and patterns of failure in patients of non-pineal supratentorial primitive neuroectodermal tumor: review of literature and clinical experience form a regional cancer center in north India. Acta Neurochir (Wien) 2015;157:1251–66. 25990846 10.1007/s00701-015-2444-2

[18] Choi SH Kim SH Shim KW Treatment outcome and prognostic molecular markers of supratentorial primitive neuroectodermal tumors. PLoS One 2016;11:e0153443. 27074032 10.1371/journal.pone.0153443 PMC4830607

[19] Picard D Miller S Hawkins CE Markers of survival and metastatic potential in childhood CNS primitive neuro-ectodermal brain tumours: an integrative genomic analysis. Lancet Oncol 2012;13:838–48. 22691720 10.1016/S1470-2045(12)70257-7 PMC3615440

[20] Richardson C RAD51, genomic stability, and tumorigenesis. Cancer Lett 2005;218:127–39. 15670890 10.1016/j.canlet.2004.08.009

